# Circulating tumor DNA profiling by next generation sequencing reveals heterogeneity of crizotinib resistance mechanisms in a gastric cancer patient with *MET* amplification

**DOI:** 10.18632/oncotarget.15457

**Published:** 2017-02-17

**Authors:** Juan Du, Xue Wu, Xiaoling Tong, Xiaonan Wang, Jia Wei, Yang Yang, Zhili Chang, Yu Mao, Yang W Shao, Baorui Liu

**Affiliations:** ^1^ The Comprehensive Cancer Centre of Drum Tower Hospital, Medical School of Nanjing University, Clinical Cancer Institute of Nanjing University, Nanjing, Jiangsu, 210008, China; ^2^ Geneseeq Technology Inc., Toronto, Ontario, M5G1L7, Canada; ^3^ Nanjing Geneseeq Technology Inc., Sino-Danish Life Science Park, Nanjing, Jiangsu, 210032, China

**Keywords:** circulating tumor DNA, next generation sequencing, MET, crizotinib, drug resistance

## Abstract

Crizotinib has been used to counter *MET* gene amplification in a number of different human malignancies. Transient response to crizotinib in *MET*-amplified gastric cancer has been reported, but the mechanisms of resistance are not well studied. Here, we reported a stage IV gastric cancer patient with high levels of *MET* amplification. The implementation of crizotinib treatment led to significant symptomatic improvement in the first 2 months, but was followed by rapid disease progression. Periodic mutation profiling of patient's circulating tumor DNA (ctDNA) by next generation sequencing (NGS) revealed a number of genetic alterations including re-occurrence of *MET* amplification, multiple secondary *MET* mutations, a dramatic increase of *FGFR2* gene relative copy number as well as mutations in other downstream and bypassing elements, which may collectively related to the patient's cancer progression. Our results illustrate the complex and heterogeneous molecular mechanisms for crizotinib resistance in this patient, and demonstrate the great potential of ctDNA profiling for treatment decision-making and prognosis in clinical practice.

## INTRODUCTION

*MET* amplification is reported to occur in approximately 5% of gastric cancer patients, and targeted drug crizotinib is currently undergoing a clinical trial of advanced *MET*-positive gastric cancer (ClinicalTrials.gov identifier: NCT02435108) [1, 2]. Furthermore, administration of crizotinib to a small cohort of esophagogastric cancer patients with *MET* amplification resulted in initial tumor shrinkage; however, cancer progression occurred within months and the mechanisms for drug resistance were not elucidated [2]. Here, we conducted targeted next generation sequencing (NGS) on the circulating tumor DNA (ctDNA) of a stage IV gastric cancer patient, and identified a reservoir of mutations that echoed the mutations found in a contemporaneous tissue biopsy including *MET* amplification. Mutation profiling of serial ctDNA samples throughout the course of crizotinib treatment uncovered a dramatic change in the genomic landscape, which could be responsible for rapid development of drug resistance and disease progression.

## RESULTS

**Figure 1:**
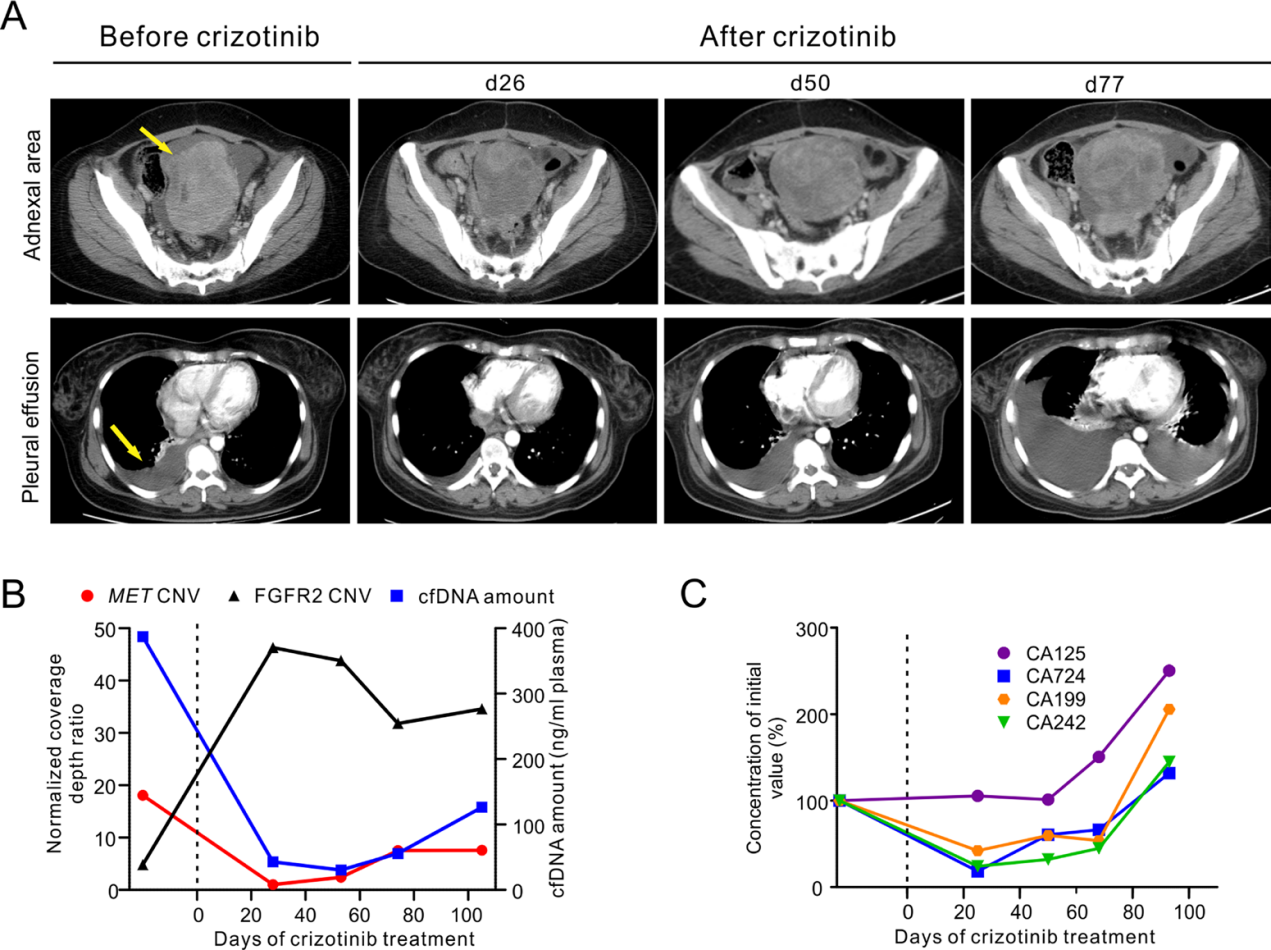
Clinical and genetic monitoring of the gastric cancer patient before and during crizotinib treatment (**A**) CT images before and during crizotinib treatment are shown at different time points to monitor metastatic tumor size in the left adnexa area (top panel) and pleural effusion (bottom panel). Yellow arrows indicate the metastatic tumor and pleural effusion before treatment. (**B**) Total cfDNA plasma concentrations, *MET* and *FGFR2* relative copy number changes in cfDNA are shown at different time points. Relative copy number was calculated as normalized coverage depth ratio to whole blood control sample. (**C**) Multiple cancer protein biomarker levels were measured at different time points. All measurements were normalized to the initial levels at diagnosis. Dotted line at Day 0 indicates the start of crizotinib administration (B, C). The time points of ctDNA were calculated from the date of starting crizotinib treatment. d, day.

Our subject was a 32-year-old female, diagnosed with stage IV signet ring cell carcinoma of the stomach (Supplementary Figure 1A), a highly malignant gastric cancer with poor prognosis [3]. At the time of diagnosis, tumors had metastasized to the bilateral adnexa of uterus (Supplementary Figure 1B) and possibly lymph nodes in greater omentum (data not shown). Multiple bone lesions were also observed by Positron Emmission Tomography-Computed Tomography (PET-CT) (Supplementary Figure 1C). The patient received a surgical resection to remove the right adnexa of uterus and partial left ovary, and was subsequently subjected to 8 cycles of chemotherapeutic treatment and 3 cycles of targeted radiation treatment (Supplementary Figure 2). However, no clinical benefits were observed and the patient showed progressive deterioration with a growing metastatic tumor size in the left adnexa area, pleural effusion (Figure 1A) and increasing bone lesions (data not shown).

Targeted NGS of 382 cancer-relevant genes and 16 genes frequently rearranged in solid tumors was performed on a tissue biopsy from her left adnexa of uterus and a contemporaneous ctDNA sample from blood plasma to identify clinically actionable mutation(s) (Supplementary Table 1). Both samples exhibited similar mutation spectra, with the most noticeable genomic alteration being an 18.1- and 17.8-fold relative copy number gain of the *MET* gene in the tissue biopsy and ctDNA, respectively (Figure 1B and Supplementary Table 2). Other common genomic abnormalities found were a relative copy number loss of *TP53* and *APC* as well as a number of inactivating mutations on tumor suppressors such as *APC*-R216X, *APC*-K1444fs, *CDKN1B*-P137fs and *TP53*-L111R (Figure 3 and Supplementary Table 2). *FGFR2* amplification was also identified in the ctDNA sample (Figure 1B). However, it was absent in the tissue biopsy suggesting that it may present in other tumor site(s). A one-year old archived FFPE tissue sample from this patient's right adnexa of uterus were also examined with the majority of these abnormalities undetectable except for the relative copy number loss of *APC* gene (Supplementary Table 2).

The patient commenced a monotherapy with crizotinib in order to target *MET* amplification. Serial ctDNA mutation profiling by targeted NGS was performed monthly to monitor tumor burden and treatment response (Supplementary Figure 2). The patient's condition improved immensely during the first month of drug administration, as evident by a marked decrease in abdominal tumor size and pleural effusion observed at day 26 post-treatment (Figure 1A). Crizotinib treatment also decreased the level of several cancer protein biomarkers, including CA724, CA199 and CA242, plasma concentration of total cell free DNA (cfDNA) and *MET* relative copy number (Figure 1B and 1C). In contrast, the relative copy number of *FGFR2* in ctDNA increased to 46.3-fold on day 28 post-treatment and maintained at such high level thereafter (Figure 1B). As crizotinib treatment continued, the concentration of plasma cfDNA and the relative copy number of *MET* began to increase, with the latter reaching 7.5-fold at day 74 (Figure 1B). Clinical parameters such as CT imaging and cancer biomarker levels also showed signs of progressive disease, and confirmed that the patient had developed drug resistance (Figure 1A and 1C).

**Figure 2:**
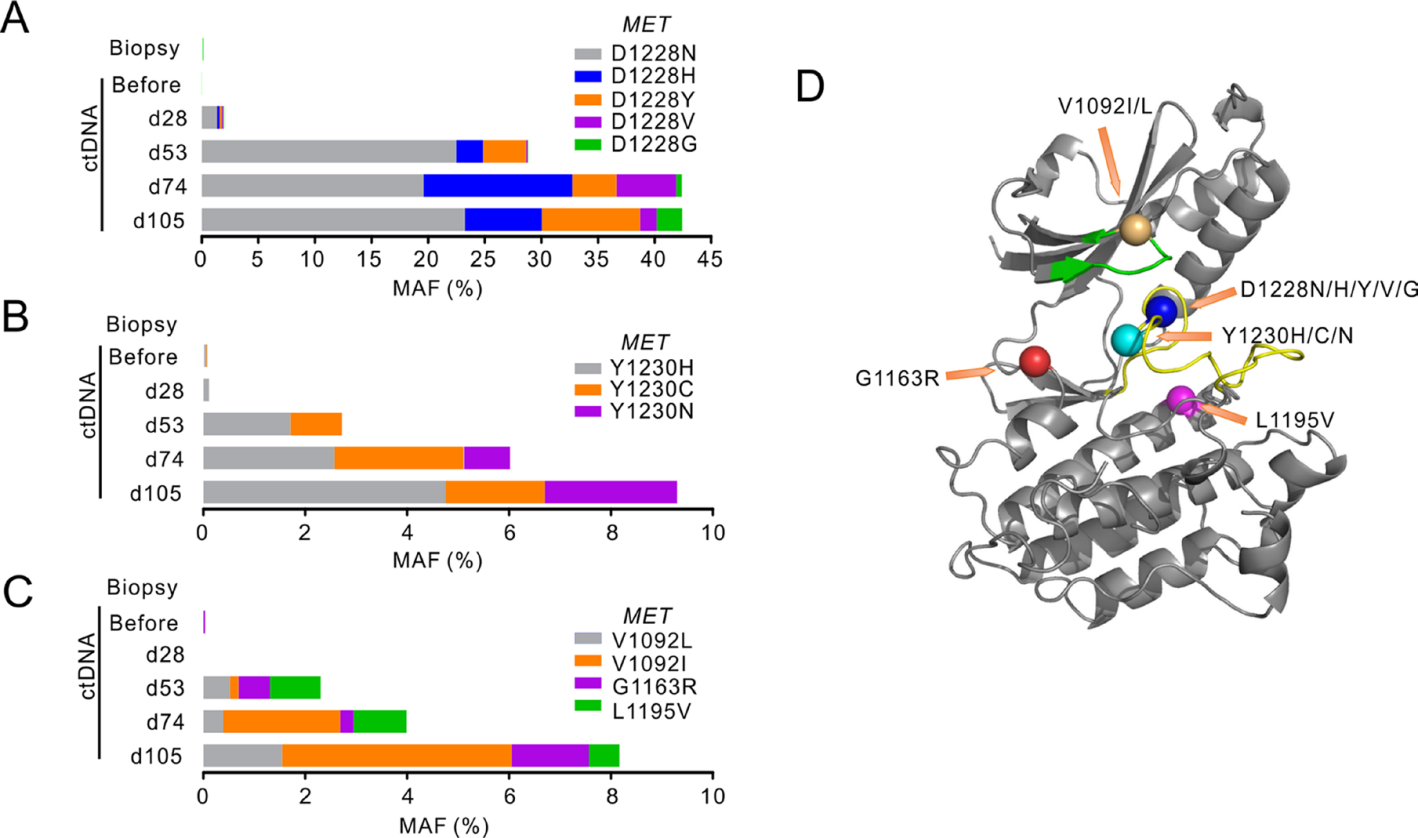
*MET* mutations identified following crizotinib treatment (**A**–**C**) The stacked bars show mutant allele frequencies (MAFs) of different mutations identified in MET tyrosine kinase (TK) domain in different sample types before and after crizotinib treatment. Mutations at MET D1228 were grouped in A, MET Y1230 mutants were grouped in B, and others were grouped in C. The time points of ctDNA were calculated from the date of starting crizotinib treatment. d, day. (**D**) Crystal structure of the MET TK domain (1R0P, RCSB Protein Data Bank) shows the localizations of all MET mutant residues identified. The green band shows ATP binding loop and the yellow band shows activation loop. Orange arrows point to the mutant residues shown as colored balls.

Interestingly, *de novo MET* mutations at D1228/Y1230 residues were detected in ctDNA as early as day 28 (Figure 2A and 2B), when CT imaging and protein biomarker levels were still showing promising response to crizotinib (Figure 1A and 1C). Sequencing of ctDNA at later dates revealed a steady rise in the variety and abundance of *MET* secondary mutations (Figure 2). A total of 8 *MET* mutations were detected on day 105 at the D1228/Y1230 positions, with D1228N and Y1230H in dominant (Figure 2A, 2B and 2D). The majority of these *MET* secondary mutations have been reported to be associated with crizotinib resistance in different cancers [4–6]. *MET* mutations at other residues in MET kinase domain were also identified, albeit at lower mutant allele frequency (MAF) (Figure 2C and 2D). This outbreak of *MET* mutations is likely due to reshaping of tumor subpopulations under the selective pressure exerted by crizotinib treatment.

**Figure 3:**
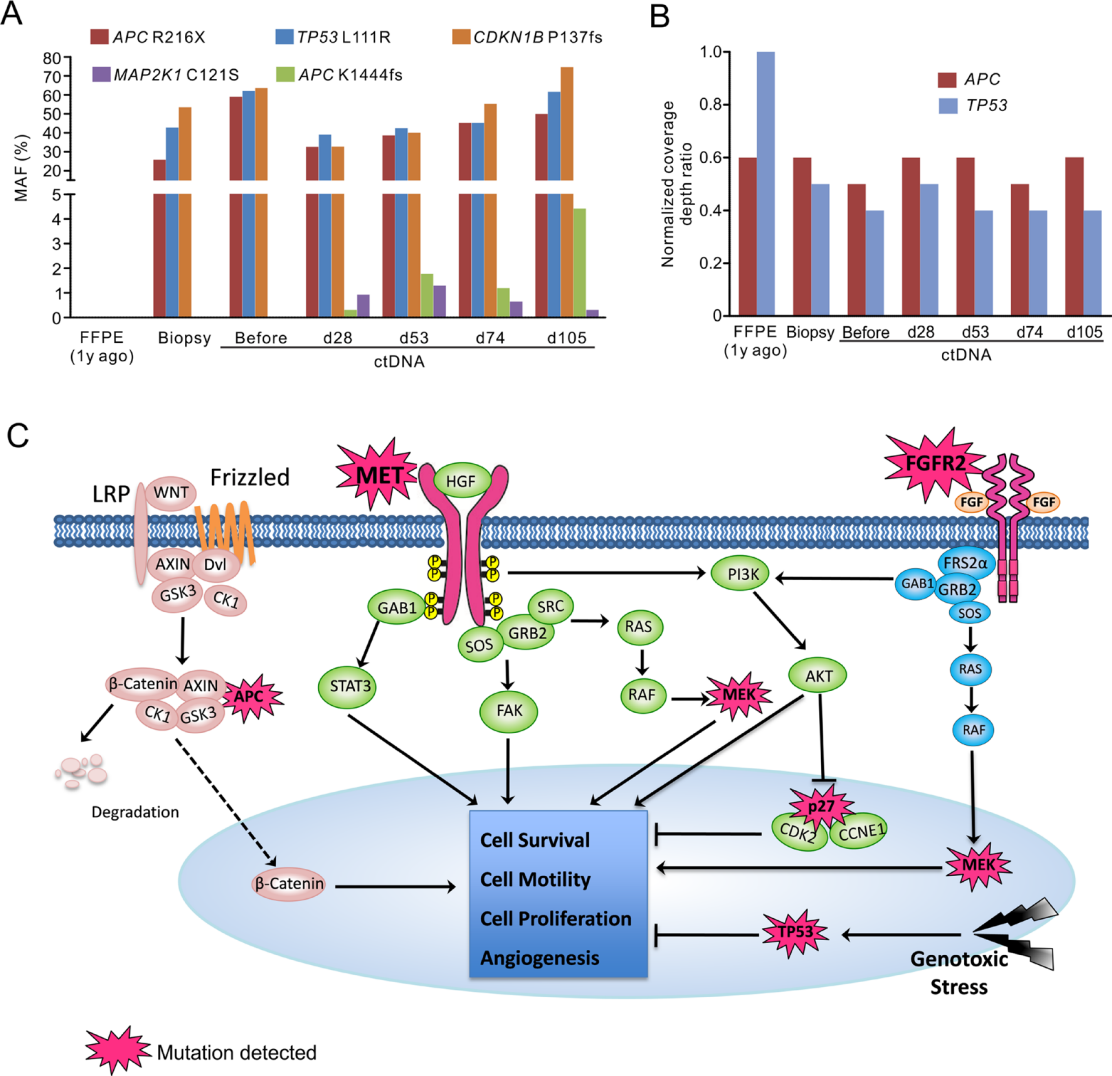
Targeted NGS with pan-cancer gene panel identified multiple genetic alterations potentially contributed to patient's drug resistance The MAFs (**A**) and relative copy number changes (**B**) in multiple genes in different sample types before and after crizotinib treatment. d, day; y, year. The time points of ctDNA were calculated from the date of starting crizotinib treatment. (**C**) Signaling pathways that were possibly influenced by mutated elements were summarized. In addition to overexpression of *MET* and *FGFR2* receptors, and *MET* activating mutations, gain-of-function of their downstream signaling (MEK1), and loss-of-function of tumor suppressors (TP53, APC and p27) may also contribute to the drug-resistance and disease progression in this patient.

In addition, a novel *MAP2K1* (MEK1) activating mutation C121S appeared in ctDNA on day 28, which functions downstream of both MET and FGFR2 pathways. Inactivating mutation *APC*-K1444fs also appeared on day 28 and its MAF increased along with disease progression (Figure 3A). Conversely, mutations in *APC*, *TP53* and *CDKN1B* (p27) identified in pre-treatment tumor only showed relatively moderate fluctuations in their MAFs (Figure 3A and 3B). Figure 3C summarized the diversity of newly acquired mutations that may be collectively responsible for crizotinib resistance in this patient.

## DISCUSSION

Crizotinib, as a potent MET inhibitor, has demonstrated promising effects in treating *MET*-amplified esophagogastric cancer [2, 7]. However, tumors experienced progression shortly after treatment [2] and the mechanisms of resistance were not clear. Comprehensive genomic profiling in this study identified *MET* amplification in the patient and led to administration of crizotinib. Serial post-treatment ctDNA monitoring revealed multiple genetic alterations that might be of value in explaining tumor recurrence after 2 months of treatment. MET D1228 and Y1230 residing in the activation loop of the kinase domain are predicted to reduce the binding affinity of crizotinib to MET and also facilitate ligand-independent activation of MET [8–10]. Mutations at V1092, G1163 and L1195 are located in the inhibitor-binding pocket of MET with possible effects in interrupting crizotinib binding [9, 11, 12]. Activation of downstream signal molecule MEK1 (*MAP2K1*-C121S) has also been reported and could bypass MET inhibition leading to drug-resistance [13].

Co-amplification of receptor tyrosine kinases, such as *HER2* or *EGFR* in *MET*-amplified esophagogastric cancer has been reported as another mechanism that drives resistance to MET inhibitors [7]. In this study, increase of *FGFR2* relative copy number was initially observed in pre-treatment ctDNA sample at low level, and then dramatically elevated throughout the treatment, indicating the potential outgrowth of *FGFR2*-amplified clones under MET-inhibition selection pressure. *FGFR2* amplification has been identified in gastric cancers and found to be associated with a poor prognosis [14, 15]. Although several FGFR inhibitors have been evaluated in clinical trials, none of them have yet been approved for clinical use [16–18]. In summary, the rapid progression of this patient might be due to both acquired resistance mutations and pre-existing molecular heterogeneity in tumor. The complexity and diversity of potential drug resistance mechanisms in this case highlight the importance of comprehensive molecular analysis for developing therapeutic strategies for this disease.

## MATERIALS AND METHODS

### Ethical compliance

Patient information and clinical samples were obtained from The Comprehensive Cancer Centre of Drum Tower Hospital. The patient has given written consent for specimen collection and the following genetic testing. Sample collection and preparation protocols were approved by the Drum Tower Hospital Ethics Committee.

### Tissue and blood sample collection

A one-year old archived FFPE block of tumor tissue from the patient's resected right adnexa of uterus and a fresh tissue biopsy from the patient's left adnex metastatic site were acquired from the pathology department of Drum Tower Hospital. Serial blood samples were collected before and during crizotinib treatment for cfDNA extraction. In brief, 5–10 ml peripheral blood was collected in an EDTA-coated tube (BD Biosciences), and plasma was prepared by centrifuging at 1800 × g for 10 minutes at 4 degree within 2 hours of blood withdrawing. Tissue and blood/plasma samples were sent to the core facility of Nanjing Geneseeq Technology Inc. (Nanjing, China) for DNA extraction and genetic testing.

### Cancer biomarker testing

Serum levels of CA125, CA199 and CA242 were measured with enzyme-linked immunosorbent assay kits (Fujirebio Diagnostics), and CA724 level was measured with electro-chemiluminescence assay kit (Roche Diagnostics) in Drum Tower Hospital's pathology laboratory. Other clinical inspections (e.g. CT, PET-CT imaging) were carried out in Drum Tower Hospital to assess the abdominal tumor burden and pleural effusion for better monitoring disease progression.

### DNA extraction and quantification

cfDNA was extracted from 3–5 ml plasma using NucleoSpin Plasma XS kit (Macherey Nagel) according to its manufacturer's protocol. Genomic DNA from the whole blood sample and fresh tissue biopsy was extracted by DNeasy Blood & Tissue kit (Qiagen). FFPE sections were de-paraffinized with xylene and subsequently subjected to DNA extraction with QIAamp DNA FFPE Tissue Kit (Qiagen). Purified DNA was qualified on Nanodrop2000 (ThermoFisher Scientific) and quantified using Qubit 2.0 (ThermoFisher Scientific). Size distribution of cfDNA was examined by Bioanalyzer 2100 (Agilent Technologies).

### Library preparation

1 μg genomic DNA from tissue biopsy, FFPE sections and whole blood were sheared into 350-bp fragments by Covaris M220 instrument (Covaris). For cfDNA, 2–100 ng DNA was used for library preparation without prior fragmentation. Sequencing libraries were constructed using KAPA Hyper Prep kit (KAPA Biosystems) according to manufacturer's protocol. In brief, DNA samples were subjected to end-repairing, A-tailing and ligation of indexed adapters, and subsequently size selection with Agencourt AMPure XP beads (Beckman Coulter). Resulted libraries were subjected to PCR amplification with numbers of PCR cycles suggested by the manufacturer's protocol according to DNA input. Amplified libraries were purified by Agencourt AMPure XP beads and quantified by Qubit 2.0.

### Library hybridization and sequencing

Libraries with different indexes were pooled together in optimized ratios to reach up to 2 μg of total DNA. Human Cot-1 DNA (Life Technologies) and xGen universal blocking oligos (Integrated DNA Technologies) were added as blocking reagents to reduce non-specific hybridization. Customized xGen lockdown probes (Integrated DNA Technologies) targeting 5,804 exons of 382 cancer-relevant genes and 37 introns of 16 fusion genes were used for hybridization enrichment (Supplementary Table 1). The capture reaction was performed with NimbleGen SeqCap EZ Hybridization and Wash Kit (Roche) and Dynabeads M-270 (Life Technologies) according to manufacturers’ protocols. Post-captured libraries were PCR amplified with Illumina p5 (5′ AAT GAT ACG GCG ACC ACC GA 3′) and p7 primers (5′ CAA GCA GAA GAC GGC ATA CGA GAT 3′) in KAPA HiFi HotStart ReadyMix (KAPA Biosystems), followed by purification with Agencourt AMPure XP beads, and quantified by qPCR method using KAPA Library Quantification kit (KAPA Biosystems). The size distribution of the library was analyzed by Bioanalyzer 2100 (Agilent Technologies). Target-enriched libraries were then sequenced on Illumina MiSeq or HiSeq4000 NGS platforms (Illumina) according to manufacturer's instructions. Targeted capture and sequencing performance of all the samples were summarized in Supplementary Table 3.

### Sequence data processing

Trimmomatic [19] was used for adapter identification and quality filtering of FASTQ files. Leading/trailing low quality (quality reading below 15) or N bases were removed. Paired-end reads from each sample were mapped to hg19 (Human Genome version 19) reference genome by Burrows-Wheeler Aligner (BWA-mem, v0.7.12) [20] with parameters (−t 8 -M). Local realignment around indels and base quality score recalibration was applied with Genome Analysis Toolkit (GATK 3.4.0) [21]. SNPs/indels callings were made by VarScan2 (minimum quality score = 15 and otherwise default parameters) to detect mutations with MAF < 10% (http://dkoboldt.github.io/varscan/) and HaplotypeCaller/UnifiedGenotyper in GATK with default parameters to detect mutations with MAF > 10%, followed by filtration against dbSNP (v137) and 1000 Genome data sets. Germline mutations were identified by comparing to its matching whole blood DNA samples. Mutations were called when their MAFs are above 1% with at least 3 high quality non-paired mutant reads (> Q30) on different strands and manually inspected in Integrative Genomics Viewer (IGV, Broad Institute). However, when a mutation was called in any of the ctDNA or tissue samples, the sequencing reads of other samples were retrospectively inspected to detect such mutation with MAF < 1%, but has at least 3 high quality non-paired mutant reads (> Q30) on different strands, and reported (Supplementary Table 2). Genomic fusions were identified by FACTERA with default parameters [22]. Copy number variations (CNVs) were detected using ADTEx (http://adtex.sourceforge.net) with default parameters. Proposed discrete wavelet transform (DWT) was used to reduce intrinsic noise of the depth of coverage (DOC) ratios. The relative copy number gain/loss of each targeted region is quantified by hidden Markov model (HMM). Germline CNVs were identified by comparing patient's blood sample to normal human HapMap DNA sample NA18535 (Coriell Institute) for each captured region (exonic region). Somatic CNVs were identified by comparing tumor/ctDNA sample to blood sample for each exon.

## SUPPLEMENTARY MATERIALS FIGURES AND TABLES


