# Supplementary material for: Circulating tumor DNA profiling by next generation sequencing reveals heterogeneity of crizotinib resistance mechanisms in a gastric cancer patient with MET amplification
Source: Oncotarget. 2017 Feb 17;8(16):26281–7. doi: 10.18632/oncotarget.15457 (PMC5432256; doi:10.18632/oncotarget.15457)
Supplement: Supplementary file 1 [file oncotarget-08-26281-s001.pdf]

Circulating tumor DNA profiling by next generation sequencing reveals heterogeneity of crizotinib resistance mechanisms in a gastric cancer patient with *MET* amplification

Supplementary Materials

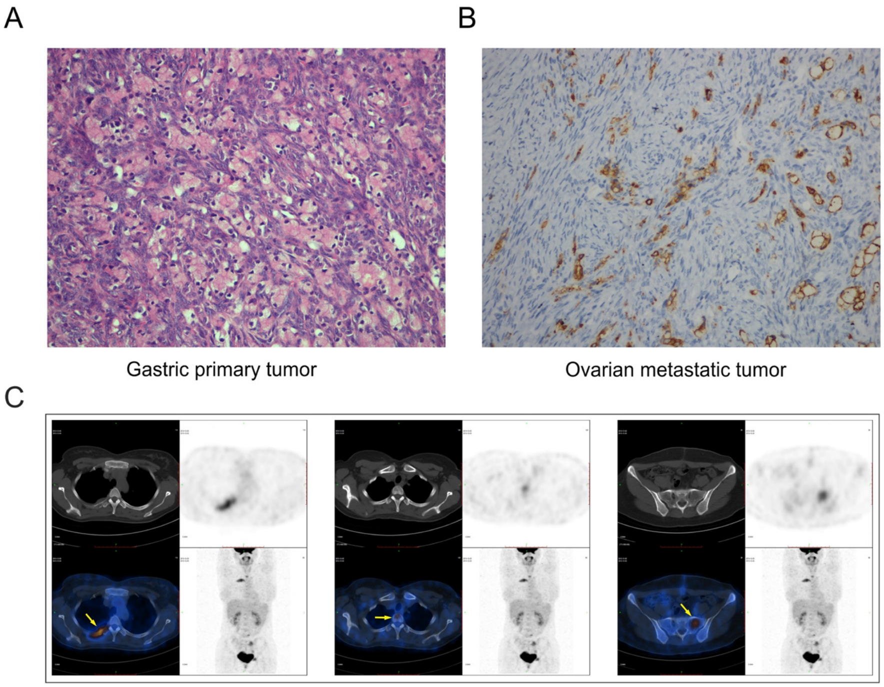

Supplementary Figure 1: Histological analysis and PET-CT images at diagnosis show metastatic disease of the patient. (**A**) H&E staining (20×) of primary tumor in stomach shows signet ring cells. (**B**) Cytokeratin staining (20×) of metastatic tumor in right adnexa. (**C**) PET-CT images show multiple bone lesions as indicated by arrows.

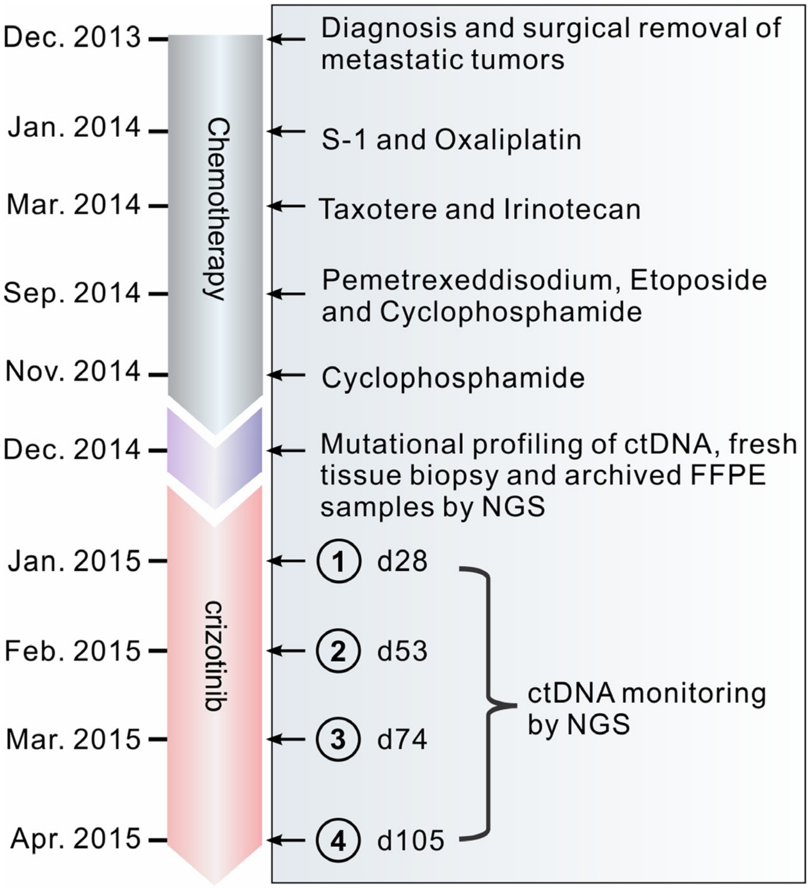

Supplementary Figure 2: Disease and treatment timeline of a stage IV gastric cancer patient. From the time of diagnosis up until November 2014, the patient received 8 cycles of chemotherapeutic treatment in combination with targeted radiotherapy on multiple occasions for the treatment of bone metastasis. Targeted NGS-based mutational profiling test was performed in December 2014 on multiple sample types, followed by periodical ctDNA genetic tests during the course of crizotinib treatment.

Supplementary Table 1: Genes covered by targeted NGS panel

ABCC2	BCL2L11	CDKN2A	DLG2	FANCF	HBA1	KDM5A	MSH6	PDGFRA	RAD51	SMC1A	TOP2A	ALK
ACTB	BLM	CDKN2B	DMNT3A	FANCG	HBA2	KDR	MTHFR	PDGFRB	RAF1	SMC3	TP53	BCL2
ADH1B	BMPR1A	CDKN2C	DNM2	FANCL	HBB	KIT	MTOR	PDK1	RARA	SMO	TP63	BCR
AIP	BRAF	CEBPA	DOT1L	FAT1	HDAC1	KMT2B	MUTYH	PHF6	RASGEF1A	SOX2	TPMT	BIRC3
AKT1	BRCA1	CEP57	DPYD	FBXO11	HDAC2	KMT2C	MYC	PHOX2B	RB1	SPOP	TRAF2	BRAF
AKT2	BRCA2	CHD4	DUSP2	FCGR2B	HDAC4	KRAS	MYCL1	PICK3R1	RECQL4	SRC	TRAF3	ETV1
AKT3	BRD4	CHEK1	EBF1	FGF19	HDAC7	LEF1	MYCN	PIK3C3	RELN	SRSF2	TRAF5	ETV5
ALDH2	BRIP1	CHEK2	ECT2L	FGFR1	HGF	LMO1	MYD88	PIK3CA	RET	STAG2	TSC1	EWSR1
ALK	BTG2	CKS1B	EED	FGFR2	HNF1A	LYN	NBN	PIK3CD	RHOA	STAT3	TSC2	KMT2A
AMER1	BTK	CREBBP	EGFR	FGFR3	HNF1B	LYST	NCSTN	PIK3R1	RICTOR	STAT5A	TSHR	MYC
AP3B1	BTLA	CRKL	EGR1	FGFR4	HRAS	LZTR1	NF1	PIK3R2	RNF43	STAT5B	TTF1	PDGFB
APC	BUB1B	CSF1R	EP300	FH	ID3	MAP2K1	NF2	PLK1	ROS1	STIL	TUBB3	RAF1
AR	C11orf30	CSF3R	EPCAM	FIP1L1	IDH1	MAP2K2	NFKBIA	PMS1	RPTOR	STK11	TYMS	RARA
ARAF	CALR	CTCF	EPHA3	FLCN	IDH2	MAP2K4	NKX2-1	PMS2	RRM1	STMN1	U2AF1	RET
ARID1A	CBL	CTLA4	ERBB2	FLT1	IGF1R	MAP3K1	NOTCH1	POLD1	RUNX1	STX11	UGT1A1	ROS1
ARID2	CCND1	CTNNB1	ERBB3	FLT3	IGF2	MCL1	NOTCH2	POLE	SBDS	STXBP2	UNC13D	TMPRSS2
ARID5B	CCNE1	CUX1	ERBB4	FLT4	IKBKE	MDM2	NPM1	POT1	SDHA	SUFU	VEGFA	
ASXL1	CCT6B	CXCR4	ERCC1	GADD45B	IKZF1	MDM4	NQO1	PPP2R1A	SDHB	SUZ12	VHL	
ATM	CD22	CYLD	ERCC2	GATA1	IKZF2	MECOM	NRAS	PRDM1	SDHC	TEK	WISP3	
ATR	CD274	CYP2B6*6	ERCC3	GATA2	IKZF3	MED12	NRG1	PRF1	SDHD	TEKT4	WRN	
ATRX	CD58	CYP2C19*2	ERCC4	GATA3	IL7R	MEF2B	NSD1	PRKAR1A	SERP2	TERC	WT1	
AURKA	CD70	CYP2C9*3	ERCC5	GATA4	INPP4B	MEN1	NT5C2	PRKCI	SETBP1	TERT	XIAP	
AURKB	CDA	CYP2D6	ESR1	GATA6	INPP5D	MET	NTRK1	PTCH1	SETD2	TET2	XPC	
AXIN1	CDC73	CYP2D6*3	ETV1	GNA11	IRF1	MGMT	PAG1	PTEN	SF3B1	TGFBR2	XPO1	
AXL	CDH1	CYP2D6*4	ETV4	GNA13	IRF2	MITF	PAK3	PTPN11	SGK1	TLE1	XRCC1	
B2M	CDK10	CYP2D6*6	EWSR1	GNAQ	IRF8	MLH1	PALB2	PTPN2	SH2D1A	TLE4	YAP1	
BAP1	CDK12	CYP3A4*4	EZH2	GNAS	JAK1	MLL	PARK2	PTPN6	SMAD2	TMPRSS2	ZAP70	
BARD1	CDK4	CYP3A5*3	FANCA	GRIN2A	JAK2	MLLT10	PAX5	PTPRO	SMAD3	TNFAIP3	ZNF217	
BCL2	CDK6	DAXX	FANCB	GRM3	JAK3	MPL	PBRM1	QKI	SMAD4	TNFRSF14	ZNF703	
BCL2L1	CDK8	DDR2	FANCC	GSTM1	JARID2	MRE11A	PC	RAC1	SMAD7	TNFRSF17	ZRSR2	
BCL2L2	CDKN1B	DHFR	FANCD2	GSTP1	JUN	MSH2	PDCD1	RAD21	SMARCA4	TNFRSF19		
BCORL1	CDKN1C	DICER1	FANCE	GSTT1	KDM2B	MSH3	PDCD1LG2	RAD50	SMARCB1	TOP1		

Grey background indicates genes targeted for fusion detection.

Supplementary Table 2: Mutations identified in FFPE, fresh tumor biopsy and serial ctDNA samples of the patient

Gene	Mutation	FFPE (1 yr)	Tissue biopsy	ctDNA-Before	ctDNA-d28	ctDNA-d53	ctDNA-d74	ctDNA-d105
APC	CNV	0.6	0.6	0.5	0.6	0.6	0.5	0.5
	K1444fs	-	-	-	0.31%*	1.77%	1.20%	4.42%
	R216X	-	25.81%	58.99%	32.61%	38.66%	45.30%	50.00%
CDKN1B	P137fs	-	53.47%	63.60%	32.72%	40.00%	55.22%	74.58%
FGFR2	CNV	-	-	4.8	16.3	43.8	31.8	34.6
IKBKE	G37S	Germline	Germline	Germline	Germline	Germline	Germline	Germline
MAP2K1	C121S	-	-	-	0.93%*	1.30%	0.65%*	0.31%*
MAP2K2	CNV	2.1	-	-	-	-	-	-
MET	CNV	-	18.1	17.8	-	2.4	7.5	7.6
	D1228G	-	0.07%*	0.02%*	0.10%*	0.10%*	5.29%	1.46%
	D1228H	-	-	-	0.21%	2.37%	13.13%	6.81%
	D1228N	0.58%*	0.02%*	-	1.35%	22.47%	19.60%	23.23%
	D1228V	-	-	-	0.10%*	0.10%*	5.29%	1.46%
	D1228Y	-	-	-	0.21%*	3.82%	3.89%	8.67%
	G1163R	-	-	0.03%*	-	0.62%*	0.26%*	1.51%
	L1195V	-	-	-	-	0.99%*	1.04%	0.59%*
	V1092I	-	-	-	-	0.17%*	2.30%	4.51%
	V1092L	-	-	-	-	0.52%*	0.39%*	1.55%
	Y1230C	-	-	0.02%*	-	1.01%	2.54%	1.96%
	Y1230H	-	-	0.04%*	0.11%*	1.71%	2.57%	4.76%
	Y1230N	-	-	-	-	-	0.90%*	2.60%
TP53	CNV	-	0.5	0.4	0.5	0.4	0.4	0.4
	L111R	7.33%	42.79%	62.06%	39.00%	42.43%	45.26%	61.64%
TSC2	S1661_ L1662delins	Germline	Germline	Germline	Germline	Germline	Germline	Germline

Somatic base substitutions and indels are presented as mutation allele frequencies (MAFs, %). Copy number variations (CNVs) are presented as fold changed. Germline mutations are labeled as “Germline”. -, mutation not detected. *, mutations with MAF < 1% were manually inspected.

Supplementary Table 3: Targeted capture and sequencing performance

Sample ID	Sample name	High quality PF reads	Alignment rate (%)	On-target rate (%)	PCR duplicates (%)	Coverage Uniformity (%)	Mean coverage depth (X)
F1412051073	Archived FFPE sample	2,549,528	98.9	86.3	9.1	89.7	269
T1412051072	Fresh tissue biopsy	1,339,280	99.7	82.8	4.4	91.4	140
B1412051076	whole blood control	983,374	99.8	82.3	5.4	92.6	97
ctDNA-B1412051076	ctDNA-Before	3,668,012	99.7	90.1	14.9	90.1	390
P1501221443	ctDNA-d28	12,839,642	99.6	84.0	29.7	91.5	668
P1502161879	ctDNA-d53	10,216,806	99.7	82.2	33.7	91.2	506
ctDNA-B1503061944	ctDNA-d74	2,871,450	99.5	88.9	12.4	91.3	320
R1504092399	ctDNA-d105	2,072,288	99.7	85.8	12.8	88.1	242

Coverage uniformity: % of targeted region > 0.2× mean coverage.
